# Screening for Cognitive Frailty Using Short Cognitive Screening Instruments: Comparison of the Chinese Versions of the MoCA and Q*mci* Screen

**DOI:** 10.3389/fpsyg.2020.00558

**Published:** 2020-04-03

**Authors:** Yangfan Xu, Yangyang Lin, Lingrong Yi, Zhao Li, Xian Li, Yuying Yu, Yuxiao Guo, Yuling Wang, Haoying Jiang, Zhuoming Chen, Anton Svendrovski, Yang Gao, D. William Molloy, Rónán O’Caoimh

**Affiliations:** ^1^Department of Rehabilitation Medicine, The Sixth Affiliated Hospital, Sun Yat-sen University, Guangzhou, China; ^2^Department of Rehabilitation Medicine, The Second Affiliated Hospital, Chongqing Medical University, Chongqing, China; ^3^Department of Rehabilitation Medicine, Mianzhu Hospital of West China Hospital, Sichuan University, Chengdu, China; ^4^Department of Rehabilitation Medicine, The Second People’s Hospital of Foshan, Foshan, China; ^5^UCL Division of Surgery and Interventional Science, London, United Kingdom; ^6^Department of Rehabilitation Medicine, The First Affiliated Hospital of Jinan University, Guangzhou, China; ^7^Department of Rehabilitation Medicine, Jilin Provincial People’s Hospital, Jilin, China; ^8^UZIK Consulting Inc., Toronto, ON, Canada; ^9^Centre for Gerontology and Rehabilitation, University College Cork, St Finbarr’s Hospital, Cork, Ireland; ^10^Department of Geriatric Medicine, Mercy University Hospital, Cork, Ireland; ^11^Clinical Sciences Institute, National University of Ireland Galway, Galway, Ireland

**Keywords:** frailty, cognitive frailty, cognitive screen, mild cognition impairment, dementia, China

## Abstract

**Background:**

Cognitive frailty describes cognitive impairment associated with physical decline. Few studies have explored whether short cognitive screens identify frailty. We examined the diagnostic accuracy of the Chinese versions of the Quick Mild Cognitive Impairment (Q*mci*-CN) screen and Montreal Cognitive Assessment (MoCA-CN) in identifying cognitive frailty.

**Methods:**

Ninety-five participants with cognitive symptoms [47 with mild cognitive impairment (MCI), 34 with subjective cognitive disorder, and 14 with dementia] were included from two outpatient rehabilitation clinics. Energy (work intensity) and physical activity levels were recorded. Cognitive frailty was diagnosed by an interdisciplinary team using the IANA/IAGG consensus criteria, stratified on the Clinical Frailty Scale (CFS). Instruments were administered sequentially and randomly by trained assessors, blind to the diagnosis.

**Results:**

The mean age of the sample was 62.6 ± 10.2 years; median CFS score was 4 ± 1 and 36 (38%) were cognitively frail. The Q*mci*-CN had similar accuracy in differentiating the non-frail from cognitively frail compared to the MoCA-CN, AUC 0.82 versus 0.74, respectively (*p* = 0.19). At its optimal cut-off (≤55/100), the Q*mci*-CN provided a sensitivity of 83% and specificity of 67% versus 91% and 51%, respectively, for the MoCA-CN (≤23/30). Neither was accurate in separating MCI from cognitive frailty but both accurately separated cognitive frailty from dementia.

**Conclusion:**

Established short cognitive screens may be useful in identifying cognitive frailty in Chinese adults with cognitive complaints but not in separating MCI from cognitive frailty. The Q*mci-*CN had similar accuracy to the MoCA-CN and a shorter administration time in this small and under-powered study, necessitating the need for adequately powered studies in different healthcare settings.

## Background

The prevalence of cognitive impairment, both mild cognitive impairment (MCI) (Ward et al., 2012) and dementia (Prince et al., 2013), are increasing worldwide and are associated with the clinical syndrome of frailty (Wallace et al., 2019), particularly its physical phenotype (Ma et al., 2019). While no consensus definition of frailty as yet exists (Rodríguez-Mañas et al., 2013), it is widely regarded as a risk state or vulnerability, predisposing to adverse healthcare outcomes (Clegg et al., 2013). Cognitive frailty is increasingly recognized as a separate clinical subtype of frailty (Sezgin et al., 2019a) closely connected to its prodrome, pre-frailty (Sezgin et al., 2019b). Cognitive impairment and frailty frequently co-exist, interacting in a complex relationship (Robertson et al., 2013; Grande et al., 2019). Frailty can predict cognitive disorders (Borges et al., 2019) and the presence of cognitive impairment improves the predictive validity and operationalization of frailty (Avila-Funes et al., 2009). Building on this, the International Academy on Nutrition and Aging (IANA) and International Association of Gerontology and Geriatrics (IAGG) recently published consensus criteria identifying cognitive frailty as the presence of physical frailty and cognitive impairment [MCI as defined by a Clinical Dementia Rating scale (CDR) score of 0.5], where dementia has been excluded (Kelaiditi et al., 2013).

The World Alzheimer Report (2015 and 2018) estimated that 58% of people with dementia live in low and middle income countries. China, as the worlds most populated country faces many challenges related to aging including high levels of dementia (Chan et al., 2013). At present the estimated prevalence of MCI is 20.8% among those aged over 65 in China (Jia et al., 2014). A recent study shows that the prevalence of cognitive frailty among Chinese community-dwellers (aged ≥ 60) is 2.3%, lower than that of frailty, pre-frailty and cognitive impairment overall (Ma et al., 2019). Many countries have suboptimal systems in place to identify the true prevalence of cognitive impairment including cognitive frailty, which confounds estimates and makes public health strategies and resource allocation to address this challenging (Prince, 2015; Patterson, 2018).

Early identification of cognitive frailty is important to facilitate personalized care for older people and the introduction of interventions that may slow onset of physical decline, impairment in activities and dementia (Morris et al., 2001; Kelaiditi et al., 2013). It may also help identify those who could benefit from complex interventions to slow onset of cognitive frailty (Apóstolo et al., 2018). Despite this, few screening instruments are available to screen for MCI and to our knowledge none that specifically identify cognitive frailty (Ruan et al., 2017). Further, it is not known if the co-existence of physical decline with cognitive symptoms may exacerbate cognitive symptoms further such that these are detectable using short cognitive screening instruments and whether this impacts on individual’s performance on testing, particularly those without functional impairment, i.e., MCI. At present, the most widely used cognitive screen for MCI is the Montreal Cognitive Assessment (MoCA). However, its specificity is poor in many studies, particularly at its recommended cut-off (Tsai et al., 2016; Breton et al., 2019). Further, it has a relatively long administration time, limiting its use in busy clinical settings in China. The Quick Mild Cognitive Impairment (Q*mci*) screen is a new, short cognitive screen designed to identify MCI (O’Caoimh et al., 2012), which is closely linked with pre-frailty (Amanzio et al., 2017; Sezgin et al., 2019b). It has not yet been translated and validated into Chinese.

Here, we adapted and translated the Chinese version of the Q*mci* screen (Q*mci*-CN) and compared its ability to distinguish cognitive frailty from (a) MCI, (b) non-frail older adults with and without dementia, and (c) other patients presenting with symptomatic memory loss. Finally, we examined its psychometric properties against the established Chinese version of MoCA (MoCA-CN).

## Materials and Methods

### Translation of the Q*mci* Screen

The Q*mci* screen has six subtests: orientation (10 points), 5-word registration (5 points), clock drawing, where a blank template is provided and patients are asked to set the time (15 points), 5-word delayed recall (20 points), verbal fluency (semantic for categories of words, e.g., animals) (20 points) and logical memory (immediate verbal recall of a short story read out loud to the patient) (30 points), giving a total score of 100 points with higher scores and a cut-off of ≥62 indicating likely normal cognition (O’Caoimh et al., 2013; O’Caoimh et al., 2017). The Q*mci* screen can be administered in less than 5 min and the test-retest reliability and diagnostic accuracy are good to excellent in different settings, see O’Caoimh and Molloy (2017) (O’Caoimh et al., 2017). It has moderate to high correlation with the Standardized Alzheimer’s Disease Assessment Scale-cognitive section (ADAS-cog), CDR and the Lawton-Brody activities of daily living scale (O’Caoimh et al., 2014). The Q*mci* screen was translated into Chinese (Mandarin) using a forward-backward translation approach using an expert panel of Chinese healthcare professionals, researchers and independent professional translators.

### Participants

Consecutive attendees consenting to be included were recruited from adults aged ≥50 years presenting with symptomatic cognitive symptoms attending general rehabilitation outpatient clinics in two hospitals in Guangzhou, China, between July and December 2017. Patients were then divided into three groups; subjective cognitive disorder (SCD), MCI, and dementia. In all, 47 had MCI, 34 had SCD and 14 dementia. Those with cognitive symptoms but found to have normal cognitive testing and no evidence of functional impairment were defined as having SCD consistent with a “medical help–seeking” group under the framework for SCD suggested by Jessen et al. (2014). As this was a convenience study conducted as part of routine care, normal controls were not included. MCI was diagnosed among those with objective memory loss, greater than was expected for their age but without loss of occupational functioning, according to the National Institute on Aging – Alzheimer’s Association workgroups diagnostic guidelines for Alzheimer’s disease (Albert et al., 2011). A diagnosis of dementia was made using DSM-IV (American Psychiatric Association [APA], 1994) and NINCDS-ADRDA (McKhann et al., 1984) criteria. Cognitive frailty was diagnosed by a consultant physician based on IANA/IAGG consensus criteria (Kelaiditi et al., 2013) in those with physical frailty and cognitive impairment but without dementia. Physical frailty was assessed clinically; self-reported energy levels including patients ability to perform tasks (work intensity) and usual physical activity levels were recorded. Cognitive frailty was stratified on the Clinical Frailty Scale (CFS), score from 1 (very fit) to 9 (terminally ill) (Rockwood et al., 2005). Those aged ≤50 or with clinical depression supported by a Geriatric Depression Scale score >5, or unable to communicate in Chinese were excluded. All participants completed a detailed neuropsychological assessment with the ADAS-cog and Mini-Mental State Examination at baseline. All signed informed consent before participating. This study received ethical approval from The Six Affiliated Hospital of Sun Yat-sen University.

### Data Collection

A consultant geriatrician, general rehabilitation physician and a speech and language therapist classified patients into diagnostic groups based on the interview and neuropsychological assessment. The Q*mci*-CN and MoCA-CN were administered by one of four trained assessors (health and social care professionals who were part of the research team) on the same day in random sequence, blind to the final diagnosis, who recorded the final scores and administration times. Alternative versions of the Q*mci*-CN and MoCA-CN were used to reduce learning effects (Cunje et al., 2007). Test administration was alternated and patients were not prompted or informed of the correct answers to the cognitive tests to avoid learning and fatigue effects and subsequent bias. To establish test–retest reliability, the same raters scored the Q*mci*-CN a second time on 59 patients within 2 weeks.

### Statistical Analysis

Descriptive statistics for cognitive tests were used to summarize sample data. The Kolmogorov–Smirnov test was used to test normality and found most data were normally distributed. Comparison between three groups was performed using one-way ANOVA with significant differences examined with Tukey’s HSD *post hoc* tests. Correlation analyses and reliability were conducted using Pearson correlation coefficients. Finally, receiver operating characteristic (ROC) curve analysis was used to measure diagnostic accuracy based on the area under the curve (AUC). ROC curves were compared using the DeLong method (DeLong et al., 1988). Excellent accuracy is defined by AUC values between 0.90 and 1.0; lower values represent reduced diagnostic accuracy with values between 0.50 and 0.60 regarded as a fail. Optimal cut-off points were identified using Youden’s Index. Sensitivity and specificity were reported for the selected cut-off points. All statistical analyses were performed using SPSS version 25, R version 3.5.0 (2018-04-23) – “Joy in Playing” and STATA version 14. A level of statistical significance of 0.05 was used for all inferential analysis. Where appropriate, 95% confidence intervals (CI) are reported.

## Results

Of those meeting inclusion criteria, 125 were invited to participate. Of these, 30 declined and one had incomplete data. The final sample included 95 patients. In total, 49% (*n* = 47) had MCI, 36% (*n* = 34) symptomatic cognitive symptoms but SCD and 15% (*n* = 14) dementia. The median CFS score of the sample included was 4 ± 1 and 36 patients (38%) were classified as having cognitive frailty. Descriptive statistics comparing those with cognitive frailty to the other patients are summarized in Table 1.

**TABLE 1:** Characteristics of patients included (*n* = 95).

Patient characteristics	Total (*n* = 95) *N* (%) or Mean ± SD [Range]	Cognitive frailty (*n* = 36) *N* (%) or Mean ± SD [Range]	Others (*n* = 59) *N* (%) or Mean ± SD [Range]
**Gender**			
Female	66 (70%)	23 (64%)	43 (73%)
Male	29 (30%)	13 (36%)	16 (27%)
Clinical Frailty Scale score	3.7 ± 1.0 [1–7]	4.0 ± 0 [4–4]	3.4 ± 1.3 [1–7]
Age (years)	62.6 ± 10.2 [50–89]	64.6 ± 10.1 [50–89]	61.4 ± 10.2 [50–85]
Education (years)	11.4 ± 5.5 [0–25]	9.8 ± 4.5 [0–17]	12.4 ± 5.9 [0–25]
Salary (Yuan)	4664 ± 2953 [0–16000]	4514 ± 2091 [1983–8000]	5016 ± 3240 [300–16000]
**Living arrangements**			
Living with family	84 (89%)	32 (89%)	52 (88%)
Living with a formal carer	6 (6%)	3 (8%)	3 (5%)
Living alone	5 (5%)	1 (3%)	4 (7%)
**Work intensity**			
Low	38 (40%)	18 (50%)	20 (34%)
Medium	36 (38%)	12 (33%)	24 (41%)
High	9 (9%)	1 (3%)	8 (13%)
Other (not provided)	12 (13%)	5 (14%)	7 (12%)
Hypertension	19 (20%)	10 (28%)	9 (15%)
Hyperglycemia	12 (13%)	3 (8%)	9 (15%)
Hyperlipemia	14 (15%)	4 (11%)	10 (17%)
Dyssomnia	31 (33%)	16 (44%)	15 (25%)
Q*mci*-CN score	51 ± 13 [6–76]	47 ± 10 [23–48]	53 ± 14 [6–65]
MoCA score	22 ± 4.8 [1–29]	21.5 ± 3 [4–27]	22 ± 5.5 [1–29]

### Cognitive Test Scoring and Administration

**FIGURE 1:**
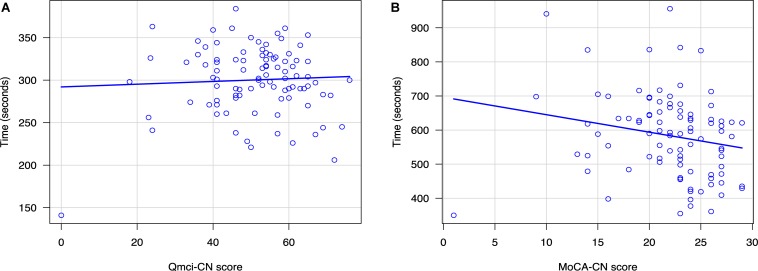
Scatterplots showing the relationship between administration time and scores on the **(A)** Chinese versions of the Quick Mild Cognitive Impairment (Q*mci*-CN) screen and **(B)** Montreal Cognitive Assessment (MoCA-CN).

We found statistically significant differences in total mean scores and standard deviation (SD) between all three diagnostic groups (SCD, MCI and dementia) for both cognitive test scores (*p*-values < 0.001). The mean scores for each diagnostic group with SD are presented in Table 2. Analyses showed that all three diagnostic groups were different from each other, with higher scores associated with higher (better) levels of cognitive ability (normal group). While no significant differences in administration times by diagnostic group were found for either the Q*mci*-CN (*p* = 0.18) or MoCA-CN *p* = 0.06), a weak gradient effect was seen with the MoCA-CN (*r* = 0.2); those with better cognition (higher scores) had non-significantly shorter administration times (see Figure 1). This was not seen for the Q*mci*-CN (*r* = 0.05). Correlation analysis, performed to examine the concurrent validity of the Q*mci*-CN showed that there was a positive, strong, statistically significant association between the Q*mci*-CN and MoCA-CN (*r* = 0.72, *p* < 0.001). Comparing test times between the two instruments, the Q*mci*-CN had a statistically significantly shorter administration time (mean 300 s, SD ± 39.6) than the MoCA-CN (mean 584 s, SD ± 124) for all participants (paired *t*(92) = 25.67, *p* < 0.001), for cognitive frailty (*p* < 0.001) and each of the three cognitive groups (all *p* < 0.001). For, cognitive frailty the difference was 272 s. For those classified as having normal cognition, the difference was 258 s; in the MCI group the difference was 289 s; In dementia the difference increased to 333 s. The Q*mci*-CN had excellent test-retest reliability (*r* = 0.92).

**TABLE 2:** Mean test scores and administration times for the Chinese versions of the Quick Mild Cognitive Impairment screen (Q*mci*-CN) and Montreal Cognitive Assessment (MoCA-CN) by diagnostic group, *n* = 95.

Cognitive test	All (*n* = 95)	SCD (*n* = 34)	MCI (*n* = 47)	Dementia (*n* = 14)	One-way ANOVA and *post hoc* tests of significance*
Q*mci*-CN score	51 ± 13	61.4 ± 7.5	48.0 ± 9.3	35.4 ± 13.9	*F*(2,91) = 41.5, *p* < 0.001
(mean ± SD)	[6–76]	[41–76]	[23–60]	[0–48]	All Tukey HSD *post hoc* tests *p* < 0.001
MoCA-CN score	22 ± 4.8	25.4 ± 2.5	21.6 ± 3.0	14.6 ± 5.3	*F*(2,91) = 54.2, *p* < 0.001
(mean ± SD)	[1–29]	[20–29]	[14–27]	[1–21]	All Tukey HSD *post hoc* tests *p* < 0.001
Q*mci*-CN screen time (seconds, mean ± SD)	300 ± 39.6	290 ± 36	306 ± 37	303 ± 53	*F*(2,91) = 1.8, *p* = 0.18
	[141–384]	[206–353]	[221–384]	[141–363]	
MoCA test time (seconds, mean ± SD)	584 ± 124	548 ± 106	595 ± 119	636 ± 159	*F*(2,90) = 2.9, *p* = 0.06
	[350–956]	[361–833]	[355–956]	[350–941]	

Reported values are Mean ± Standard Deviation (SD) [Range], MCI = Mild Cognitive Impairment; SCD = Subjective Cognitive Disorder. * Comparison between scores for SCD, MCI and dementia.

### Screening for Cognitive Frailty

**FIGURE 2:**
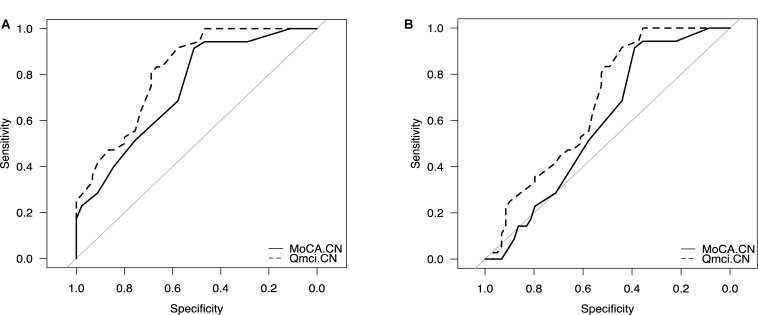
Receiver Operating Characteristic (ROC) curve analysis comparing the Chinese versions of the Quick Mild Cognitive Impairment (Q*mci*-CN) screen and Montreal Cognitive Assessment (MoCA-CN) in identifying **(A)** cognitive frailty from non-frailty and **(B)** cognitive frailty from other patients presenting with symptomatic memory loss.

Examining the accuracy of these instruments in differentiating cognitive frailty from those with MCI but without physical frailty, showed that both the Q*mci*-CN and MoCA-CN were poor at differentiating CF from MCI, AUC’s of 0.63 versus 0.51 (*p* = 0.38), respectively. Both instruments were accurate in separating cognitive frailty from dementia with the MoCA-CN having borderline but not statistically greater accuracy than the Q*mci*-CN, AUC of 0.89 versus 0.76 (*p* = 0.05), respectively. Examining the diagnostic accuracy of both screening instruments in separating those with cognitive frailty from patients who were non-frail (i.e., those with MCI but without physical frailty and SCD who were clinically robust with a CFS score <4), again showed that the Q*mci*-CN and MoCA-CN had similar, AUC 0.81 (95% CI: 0.72–0.90) versus 0.74 (95% CI: 0.63–0.85), respectively, a non-statistically significant difference (*p* = 0.19). Neither instrument was useful in distinguishing cognitive frailty from all of the other patients presenting with symptomatic memory loss (i.e., those with SCD, MCI without frailty and those with dementia); the Q*mci*-CN had an AUC of 0.68 (95% CI: 0.57–0.78) versus 0.59 (95% CI: 0.48–0.70) for the MoCA-CN (*p* = 0.10). At its optimal cut-off (≤55/100), the Q*mci*-CN had a sensitivity of 83% and specificity of 67% for differentiating cognitive frailty from the non-frail. This compared to a sensitivity and specificity of versus 91 and 51%, respectively, for the MoCA-CN at its optimal cut-off in this sample, ≤23/30. These are presented in Figure 2 and Table 3.

**TABLE 3:** Area under the curve (AUC) values and cut-offs for the Chinese versions of the Quick Mild Cognitive Impairment (Q*mci*-CN) screen and Montreal Cognitive Assessment (MoCA-CN).

Diagnostic classification	Cognitive screen	AUC [95% CI]	Comparison of AUC	Optimal cut-off point Sensitivity and Specificity
Cognitive frailty vs. Non-frail	Q*mci*-CN	0.81 [0.72–0.90]	*p* = 0.19	≤55; Sensitivity = 83%, Specificity = 67%
	MoCA-CN			
		0.74 [0.63–0.85]		≤23; Sensitivity = 91%, Specificity = 51%
Cognitive frailty vs. Other	Q*mci*-CN	0.68 [0.57–0.78]	*p* = 0.10	≤58; Sensitivity = 92%, Specificity = 44%
	MoCA-CN			
		0.59 [0.48–0.70]		≤ 24; Sensitivity = 91%, Specificity = 39%
Cognitive frailty vs. MCI without frailty	Q*mci*-CN	0.63 [0.43–0.80]	*p* = 0.38	≤58; Sensitivity = 92%, Specificity = 33%
	MoCA-CN			
		0.51 [0.34–0.67]		≤24; Sensitivity = 31%, Specificity = 83%
Cognitive frailty vs. Dementia	Q*mci*-CN	0.76 [0.63–0.90]	*p* = 0.05	≤50; Sensitivity = 53%, Specificity = 100%
	MoCA-CN			
		0.89 [0.80–0.98]		≤21; Sensitivity = 71%, Specificity = 93%
MCI/Dementia vs. SCD	Q*mci*-CN	0.91 [0.84–0.97]	*p* = 0.42	≤55; Sensitivity = 83%, Specificity = 82%
	MoCA-CN	0.87 [0.80–0.95]		≤24; Sensitivity = 95%, Specificity = 68%
Dementia vs. MCI/SCD	Q*mci*-CN	0.87 [0.80–0.95]	*p* = 0.06	≤48; Sensitivity = 100%, Specificity = 72%
	MoCA-CN	0.94 [0.89–0.99]		≤21; Sensitivity = 100%, Specificity = 73%
MCI vs. SCD	Q*mci*-CN	0.88 [0.81–0.96]	*p* = 0.39	≤60; Sensitivity = 100%, Specificity = 62%
	MoCA-CN	0.84 [0.75–0.93]		≤25; Sensitivity = 96%, Specificity = 62%
Dementia vs. SCD	Q*mci*-CN	0.99 [0.96–1.00]	*p* = 0.74	≤48; Sensitivity = 100%, Specificity = 97%
	MoCA-CN	0.99 [0.97–1.00]		≤21; Sensitivity = 100%, Specificity = 91%
Dementia vs. MCI	Q*mci*-CN	0.79 [0.67–0.91]	*p* = 0.045	≤46; Sensitivity = 93%, Specificity = 61%
	MoCA-CN	0.91 [0.83–0.98]		≤20; Sensitivity = 93%, Specificity = 74%

### Screening for Cognitive Impairment (MCI and Dementia)

**FIGURE 3:**
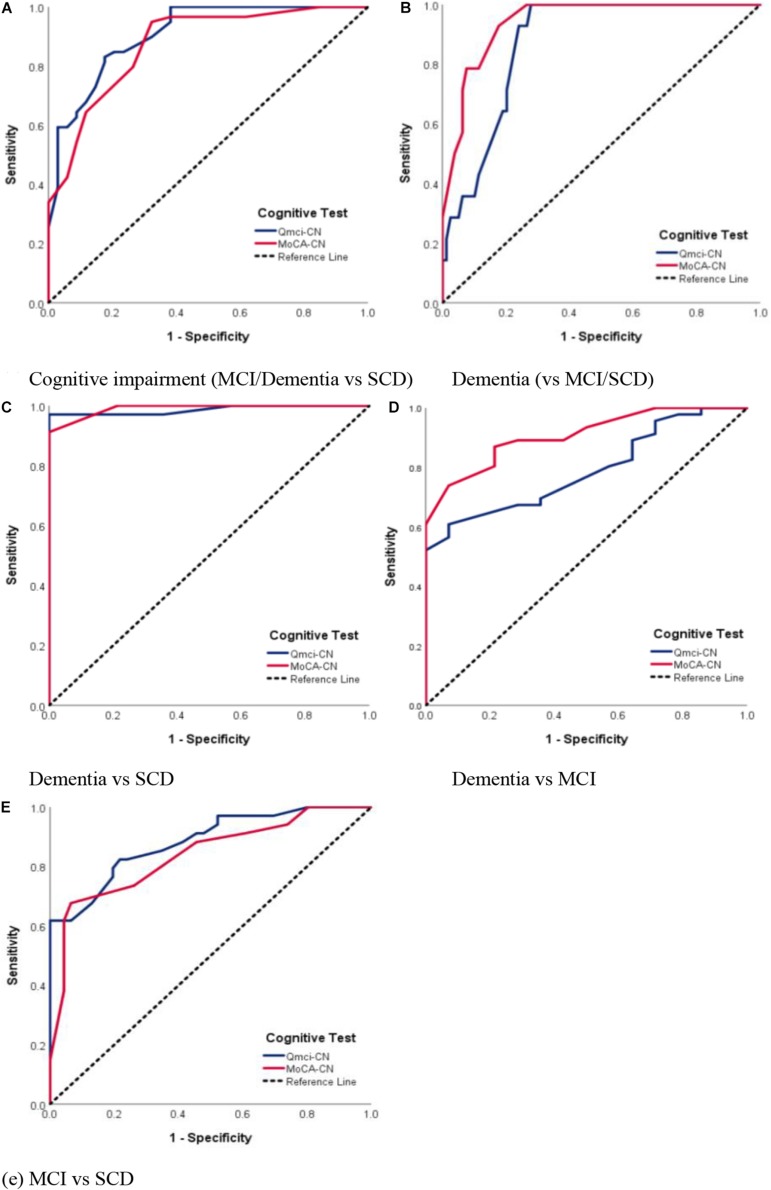
Receiver Operating Characteristic (ROC) curve analysis comparing the Chinese versions of the Quick Mild Cognitive Impairment (Q*mci*-CN) screen and Montreal Cognitive Assessment (MoCA-CN) in separating subjective cognitive disorder (SCD), mild cognitive impairment (MCI) and dementia. **(A)** Cognitive impairment (MCI/Dementia vs. SCD). **(B)** Dementia (vs. MCI/SCD). **(C)** Dementia vs. SCD. **(D)** Dementia vs. MCI. **(E)** MCI vs. SCD.

Receiver operating characteristic curve analyses were then performed to explore the ability of each cognitive test to differentiate between SCD, MCI, and dementia. This showed that both instruments had similar accuracy in separating cognitive impairment (MCI/Dementia) from normal cognition (*p* = 0.42); the Q*mci*-CN had an AUC 0.91 compared to an AUC 0.87 for the MoCA-CN. The Q*mci*-CN had a better balance in sensitivity and specificity at the optimal cut-off score of ≤55 (Sensitivity = 82%, Specificity = 83%) versus the MoCA-CN, which had a higher sensitivity (95%) but lower specificity (68%) at a cut-off of ≤24. ROC analysis showed that both instruments had similar (non-significantly different) accuracy in identifying people with dementia, AUC of 0.94 compared with AUC of 0.87 for the MoCA-CN and Q*mci*-CN, respectively. The MoCA-CN was more accurate in its predictive ability for dementia versus MCI (AUC 0.91) compared to the Q*mci*-CN (AUC of 0.79), a statistically significant difference, *p* = 0.045. These are presented in Figure 3 and Table 2.

## Discussion

Here, we explore the ability of short cognitive screening instruments to identify cognitive frailty as defined by the IANA/IAGG consensus criteria (Kelaiditi et al., 2013), showing that while both the newly translated Q*mci*-CN and established MoCA-CN are able to differentiate cognitive frailty from non-frail individuals and those with dementia, neither instrument was accurate in separating MCI from cognitive frailty in an outpatient rehabilitation setting in China. This suggests that although able to separate cognitive frailty from dementia, where physical symptoms frequently accompany cognitive decline (Tolppanen et al., 2015), the presence of physical frailty in addition to cognitive symptoms in those with normal function (i.e., MCI) does not appear to register on short cognitive screens. The Q*mci*-CN nevertheless compared favorably with the MoCA-CN, with no statistically significant difference in their diagnostic accuracy. We also examined the diagnostic accuracy of the Q*mci*-CN against the MoCA-CN in separating those presenting with cognitive complaints, showing that the Q*mci*-CN’s ability to distinguish MCI from SCD or dementia in this sample was good to excellent but that the time taken to complete it was significantly shorter, which is particularly convenient in a rehabilitation clinic setting. The MoCA-CN was significantly better able to separate MCI from dementia. The Q*mci*-CN represents another external validation of the instrument, after the Irish, Dutch, Australian, Turkish, Italian, Taiwanese, Japanese, and Portuguese versions (Bunt et al., 2015; O’Caoimh et al., 2016; Clarnette et al., 2017; Yavuz et al., 2017; dos Santos et al., 2019; Iavarone et al., 2019; Lee et al., 2018; Morita et al., 2019). This study adds more evidence to support its use in patients with MCI in busy clinical setting.

Although, the results did not show that the Q*mci*-CN is superior at differentiating cognitive frailty, it is likely that it would have been underpowered to show this; based on previous studies comparing the Q*mci* screen to the MoCA a sample of 300 patients with MCI and 300 controls would be required (O’Caoimh et al., 2016). Due to time and resource constraints recruitment was discontinued after 6 months. Further, because of this additional research is needed to come to any confident conclusions regarding diagnostic accuracy. Nevertheless, its administration took significantly less time than the MoCA-CN (*p* < 0.001) and no marked gradient effect was evident compared to that seen for the MoCA-CN, where people with dementia took much longer to complete the test. The Q*mci*-CN took on average 300s (5 min) to complete, while the MoCA-CN took on average 584s (9.7 min), almost double the time. Given this, the Q*mci*-CN appears to be more convenient to use in clinical settings where time is limited or numbers attending high, which is especially relevant in China. Additional research is also required to examine if this time saving could improve efficiency (e.g., more patients seen per clinic) or if there are cost savings associated with the reduced administration time of the Q*mci*-CN versus the MoCA-CN.

This paper also provides the optimal cut-off scores for both instruments to identify cognitive frailty, which are similar to those for identifying cognitive impairment in this sample, particularly MCI suggesting that there is likely to be significant overlap between these patients (Won et al., 2018). This is reinforced by the fact that both instruments were poor at separating MCI from cognitive frailty. Their diagnostic accuracy was better at distinguishing cognitive frailty from dementia, i.e., those with cognitive impairment with physical impairment and functional impairment, respectively. For example, the optimal cut-off for the Q*mci*-CN in separating MCI from normal was ≤60, similar to that found in an Irish cohort (O’Caoimh et al., 2016). Cut-off scores for dementia were however, lower than those found in other countries. Possible reasons for this discrepancy include the lower level of education of participants, a mean/median of 11 versus 12 years in the studies in Ireland and Canada, and the setting as all participants were recruited from rehabilitation clinics. At the same time, the MoCA-CN’s optimal cutoff score for cognitive impairment was ≤24 (Sensitivity = 95%, Specificity = 68%), which is also lower than the recommended MoCA cutoff score (< 26) (Nasreddine et al., 2005).

### Limitations

First, we cannot be certain that all patients were classified appropriately, as differentiating cognitive frailty from MCI and from dementia with frailty was based on clinical criteria, which are inherently subjective. Nevertheless, within the confines of these criteria, patients were correctly classified. Further, the neuropsychological testing used here is different to that applied in IANA/IAGG criteria for cognitive frailty (i.e., the CDR). This said there is still no gold standard to diagnose cognitive frailty and detailed neuropsychological testing (i.e., ADAS-cog), which are routine in our clinics was conducted. Further, IANA/IAGG have been criticized for being impractical in busy clinical practice (Won et al., 2018). Second, the sample size was small, especially the number of people with cognitive frailty (*n* = 36) such that the sample was not powered adequately to detect significant differences in the diagnostic accuracy of the instruments. This is particularly evident in the analysis examining the performance of the screening instruments in separating MCI from cognitive frailty with only 12 patients with MCI without physical frailty available. The low accuracy for this comparison raises the concern that the instruments are not diagnosing cognitive frailty specifically, but just performing as would be expected in separating people with normal and abnormal cognition regardless of physical ability. This requires a larger sample to evaluate. Third, this was a highly selected sample with those found to have clinical depression and those with atypical presentations excluded as they often present with exaggerated functional and cognitive impairments. Fourth, given the relatively homogenous sample, spectrum bias may have occurred further limiting the results (Chopard et al., 2015). Finally, cognitively healthy (asymptomatic age-matched with normal neuropsychological testing) controls were not included in this analysis. To correctly interpret the tests, particularly the psychometric evaluation of these CSIs, a control group without subjective memory problems is needed as a comparison group. This is also important as those with SCD have a higher risk for conversion to subsequent MCI and dementia, though the majority do not develop progressive cognitive decline (Jessen et al., 2020). As many studies include both groups this is needed to improve comparability with other studies.

## Conclusion

In conclusion, screening for cognitive frailty was possible using short cognitive screening instruments in this sample of middle-aged and older Chinese adults. The Q*mci-*CN screen, which is validated here for the first time in Chinese among those presenting with cognitive symptoms, appears to be a short, and reliable instrument that can be used to differentiate SCD from MCI and dementia. Here it shows similar accuracy to the MoCA-CN with a shorter administration time and can be applied in busy rehabilitation settings. While both screens separated cognitive frailty from physically robust patients and those with dementia, neither accurately separated MCI from cognitive frailty. This suggests that in this sample, as might be expected, cognitive screening instruments are better able to detect the cognitive rather physical aspects of frailty in those with cognitive decline. Further research is required to examine this and to recruit more patients to adequately power a study to investigate if short cognitive screens can accurately identify cognitive frailty in a range of different settings, such as community, memory clinics and acute hospitals in comparison with non-frail and asymptomatic normal controls. Similarly, there is a need to examine the psychometric properties of the Q*mci*-CN in more detail and compare its diagnostic accuracy to the MoCA-CN in older Chinese adults presenting with cognitive symptoms.

## Data Availability Statement

The datasets generated for this study are available on request to the corresponding author.

## Ethics Statement

All signed informed consent before participating in our research. This study received ethical approval from The Six Affiliated Hospital of Sun Yat-sen University.

## Author Contributions

YX: design, concept, data collection, analysis, writing and revising manuscript. YW: design, concept and supervision. YL, LY, ZL, XL, YY, YG, HJ, and ZC: concept and data collection. AS: statistical analysis and supervision of statistics. YG: design. DM: design, concept. RO’C: design, concept, analysis, writing, and revising manuscript.

## Conflict of Interest

DM and RO’C are copyright holders of the Q*mci* screen. AS is the owner of the UZIK Consulting Inc., Toronto, ON, Canada. The remaining authors declare that the research was conducted in the absence of any commercial or financial relationships that could be construed as a potential conflict of interest.
